# Socioeconomic Status in Adolescents: A Study of Its Relationship with Overweight and Obesity and Influence on Social Network Configuration

**DOI:** 10.3390/ijerph15092014

**Published:** 2018-09-15

**Authors:** Natalia Arias, María Dolores Calvo, José Alberto Benítez-Andrades, María José Álvarez, Beatriz Alonso-Cortés, Carmen Benavides

**Affiliations:** 1 SALBIS Research Group, Department of Nursing and Physiotherapy Health Science School, University of León, Avenida Astorga s/n, Ponferrada 24401, León, Spain; mj.alvarez@unileon.es (M.J.A.); balof@unileon.es (B.A.-C.); 2 Administrative Law, Law Faculty, University of Salamanca, Salamanca 37008, Spain; mdcalvo@usal.es; 3 SALBIS Research Group, Department of Electric, Systems and Automatics Engineering, University of León, Campus of Vegazana s/n, León, 24071, León, Spain; jbena@unileon.es (J.A.B.-A.); mcbenc@unileon.es (C.B.)

**Keywords:** obesity, adolescent, social networks, socioeconomic status, peers

## Abstract

Socioeconomic status (SES) influences all the determinants of health, conditioning health throughout life. The aim of the present study was to explore the relationship between socioeconomic status and obesity in adolescence through an analysis of the patterns of contact between peers as a function of this parameter. A cross-sectional study was performed, analyzing a sample of 235 students aged 14 to 18 and 11 class networks. Social network analysis was used to analyze structural variables of centrality from a sociocentric perspective. We found that adolescents with a medium-low SES presented a two-fold higher probability of being overweight, but we did not detect any differences in the configuration of their social networks when compared with those of normal-weight adolescents. However, we did find significant differences in the formation of networks according to SES in the overall sample and disaggregated by gender, whereby adolescents with a high SES in general presented a higher capacity to form wider social networks. Elucidating the relationship between SES and overweight and its influence on social network formation can contribute to the design of preventative strategies against overweight and obesity in adolescents, since their social environment can provide them with several resources to combat excess weight.

## 1. Introduction

In 1974, the Canadian Minister Marc Lalonde published a pivotal report in public health that stressed the importance of health promotion and included aspects rarely considered before then, such as the environment and lifestyle [1]. Socioeconomic status (SES) conditions all the determinants of health defined by Lalonde, constraining or shaping our beliefs, behaviors and even our biology [2]. It is also at the root of health inequalities, since political, economic and social factors give rise to an unequal distribution of opportunities to enjoy health [3]. Social inequalities in childhood can lead to the same inequalities in adulthood, fueling a harmful legacy from generation to generation [4].

SES is therefore one of the many factors that can determine the existence of overweight in children, adolescents and adults. According to the World Health Organisation (WHO), it is low- and middle-income countries that are primarily affected, and the prevalence of overweight and obesity in preschool children living in countries with emerging economies can exceed 30% [5]. Numerous studies have reported an inversely proportional relationship between SES and overweight and associated problems in childhood, whereby the lower the SES, the higher the prevalence of health problems [6,7,8,9,10,11,12]. An association has also been found between SES and the two decisive factors in this major epidemic of overweight and obesity: diet and physical activity. For example, young people from certain ethnic groups with a low SES consume more fatty and high calorie foods [12] and in general have worse eating habits [13,14]. Meanwhile, the relationship between SES and physical exercise is directly proportional: the lower the former, the less the latter is performed [15].

In relation to the subject of the present study, adolescents’ personal and individual social networks determine behavior related to food and physical exercise in this age group: it has been shown that besides adults, peers and friends are also able to modify habits related to excess weight [16,17,18,19,20,21,22,23]. Although this influence on such habits can be negative as well as positive, these social networks should nevertheless be viewed as a source of material, personal and/or institutional resources from which to obtain the necessary information, support and services [24]. Adolescents need to belong to a social network with which they identify to attain satisfactory physical, psychological, and social development [25], since their social environment provides them with the tools necessary for managing group relations [26].

SES also determines the structure and function of social networks. For example, it has been demonstrated that adults with a low SES tend to report fewer social networks and less support [27]. The configuration of adolescents’ social networks might be similarly affected by this factor, compromising the support networks can provide to combat problems such as overweight and obesity, and reducing the resources available for information or support on issues related to food or physical exercise. In light of the above, our goal was to study the relationship between SES and overweight (overweight + obesity) in our sample and to relate this parameter to adolescents’ centrality in their social network at school, both in general and in relation to their weight status, by conducting a social network analysis (SNA) from a sociocentric or global perspective. We believe that the information obtained would be useful for the design of strategies to combat overweight and obesity and would shed light on one of the most pressing public health problems today: obesity in the adolescent population.

## 2. Materials and Methods 

### 2.1. Population and Sample 

We invited 776 students in their third and fourth years of compulsory secondary education at five schools in the city of Ponferrada (Spain) to participate in the study. Permission for data collection was sought from the Castile and León Education Department and the Spanish Data Protection Agency, and interviews were conducted with school heads and teachers to obtain their collaboration in the study. To participate in the study, students were asked to provide their informed consent via a form signed by their parents and designed in line with the recommendations of the University of Salamanca Bioethics Committee. This gave a detailed explanation of the purpose of the study and information on data collection and processing, pursuant to the Law on the Protection of Personal Data [28]. Participants were clearly informed that they could retract their consent once their parents had signed the form, without needing to provide a reason, and an email contact address was given should they require any further information. Participation was voluntary, and subject availability was respected at all times.

To obtain a satisfactory sample, we required a minimum participation rate of between 40 and 50% of class members. We received a response from 276 students from 11 different classes (Table 1). Weight status formed an inclusion criterion: we sought the exclusive participation of individuals classified as “normal weight”, “overweight” or “obese” according to WHO criteria [29]; hence, we excluded students classified as “low weight”. This yielded a final sample of 235 students divided into 11 networks (Table 2).

### 2.2. Data Collection 

Data were collected on gender, SES, anthropometric measurements (weight and height) and contacts in participants’ social networks at school between March and December 2015. Nursing staff trained in this procedure, collected the study data in paper-based survey and took physical measurements of the anthropometric parameters. In line with the recommendation of school heads and teaching staff, questionnaires were administered during tutorial classes and weight and size measurements were taken during physical education classes. This latter procedure required a closed changing room, a portable Seca 700 stadiometer (Seca, Hanover, MD 21076, USA) provided by the Nursing and Physiotherapy Department of the University of León and electronic Fagor Slim scales (Fagor, Mondragón (Gipuzkoa), Spain) calibrated to zero for each measurement. 

### 2.3. Variables

Gender was considered a dichotomous variable. SES (independent variable) was evaluated using the FAS II questionnaire [30,31], which assesses the family’s purchasing power according to the everyday goods purchased. Responses to the FAS II questionnaire were cored between 0 and 3: negative responses were awarded a 0, and this value rises as the number of possibilities increases. The score for the total scale ranges from 0 to 9 and was subsequently grouped into three categories that reflect socioeconomic status. The FAS II has been validated by Boyce et al. and in line with their interpretation criteria, we classified scores 0, 1 and 2 as indicating low SES; 3, 4, and 5 as medium SES; and 6 as high SES [30]. Since an initial statistical analysis indicated that only a small number of participants presented a low SES, we aggregated the two lower levels to form a medium-low SES group, thus creating a dichotomous variable consisting of high and medium-low SES.

Once each participant’s weight and size data had been collected, we calculated the percentile and body mass index (BMI) according to exact age and gender using the WHO’s Anthro Plus® application (World Health Organization, Cyberjaya, Selangor, Malaysia) [32]. Participants were then classified according to their weight status as “normal weight”, “overweight” or “obese”. Next, we generated a dichotomized variable using normal weight as the reference category and combined overweight and obesity as the second category (to represent excess weight), which we termed overweight. 

To obtain data about social network contacts (dependent variable), each questionnaire contained a closed list with the names and surnames of other classmates participating in the study, and the following question: “Using the list below, indicate how much time you spend with your classmates”, formulated in line with the recommendations proposed by other experts in SNA [18,33]. Since the definition of “peers, classmates or friends” is complex and could seriously affect estimation of its effects when completing the questionnaire [34], we assessed contact intensity by means of time frequencies, using a 5-point Likert scale where 1 = “we never spend time together” and 5 = “we’re always together” [26,35]. Please note that from the outset, all personal information that could identify any of the participants was encoded using a simulated name to ensure confidentiality.

Peer contact data were used to generate an initial n × n matrix (single-mode or type I network), consisting of students belonging to each class network. Since we wished to study contact intensity, each frequency was assigned a score, and three different adjacency matrices (0/1) were created from the initial matrix, based on three dichotomization criteria: (i) a “minimum contact” matrix, an adjacency matrix where the original value of 1 (“we never spend time together”) represented the absence of contact (0) and the values 2, 3, 4 and 5 (“we sometimes spend time together”, “we spend quite a lot of time together”, “we’re almost always together” and “we’re always together”) indicated the existence of the same (1); (ii) an “intermediate contact” matrix, where the values 1 and 2 (“we never spend time together” and “we sometimes spend time together”) indicated the absence of contact (0) and the values 3, 4 and 5 (“we spend quite a lot of time together”, “we’re almost always together” and “we’re always together”) represented the existence of a tie (1); and (iii) a “maximum contact” or “friendship” matrix, where the values 1, 2 and 3 (“we never spend time together”, “we sometimes spend time together” and “we spend quite a lot of time together”) indicated a lack of contact (0), and 4 and 5 (“we’re almost always together” and “we’re always together”) represented the existence of a relationship (1). 

For each contact intensity matrix, an analysis was conducted of the seven parameters representing social network centrality [36] from a sociocentric or global perspective: (i) outdegree (nominations emitted); (ii) indegree (nominations received); (iii) degree (number of ties that one actor has) [36]; (iv–v) closeness (in/outcloseness) (number of steps that one actor must take to reach another) [37,38]; (vi) betweenness (degree of connections that pass through an actor for one actor to reach another) [37,38]; and (vii) the eigenvector (a measure to identify the most central actors with the shortest distance to the rest of the nodes) [39]. This analysis yielded 21 normalized variables (values in which the ends were relativized) organized dichotomously according to the median for each parameter.

### 2.4. Statistical Analysis

The relationship between SES and the study variables was determined by unconditional logistic regression. In each case, we calculated the odds ratio (OR) with a confidence interval (CI) of 95%. Statistical significance (*p*-value) was established as *p* ≤ 0.05. Statistical analyses were performed using SPSS v.23 IBM, Armonk, NY 10504, USA) and network contact data were calculated using UCINET v.6.365 (Analytic Technologies, Inc, Collegeville, PA 19426, USA ) [40]. 

## 3. Results

**Figure 1:**
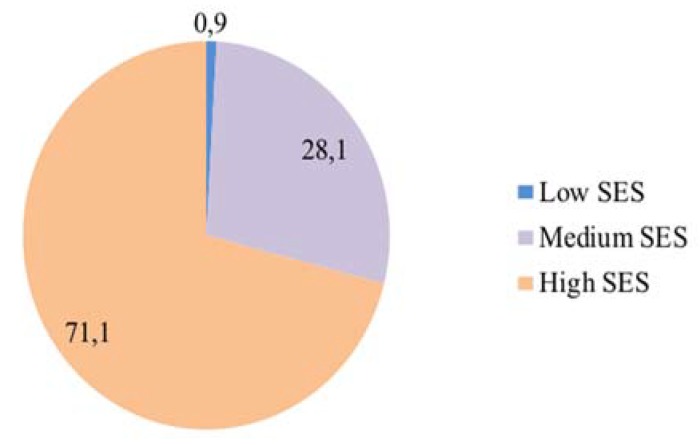
Distribution by SES in our student sample.

Descriptive data for the sample indicated that 49.4% were female (*n* = 116) and 50.6% were male (*n* = 119). Participants’ ages ranged from 14.0 to 18.1 years old, with a mean age of 15.5 ± 0.9 years old. Mean BMI was 22.1 ± 2.9 kg/m^2^ and the mean percentile value was 64.8 ± 24.1. In line with WHO criteria [29], the prevalence of overweight was 25.5% and of obesity 4.7%, indicating a total prevalence of overweight of 30.2%. Regarding SES, the mean score obtained using the FAS II questionnaire was 6.3 ± 1.6, corresponding to a high SES. As can be seen in Figure 1, 29% of the adolescents presented a medium-low SES and 71.1% a high SES. An analysis of the relationship between SES and overweight indicated that students with a medium-low SES presented a two-fold higher probability of being overweight (OR: 2.43; 95% CI: 1.33–4.40; *p* = 0.003).

**Figure 2:**
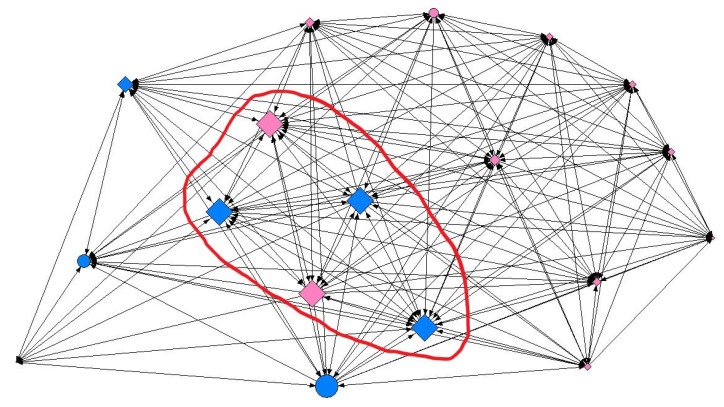
Graphical representation of one of the networks in the study at the minimum contact intensity level, where node size indicates the capacity for intermediation (betweenness). Females are shown in pink, males in blue; circles represent individuals with a medium-low SES and diamonds a high SES. Graphs were produced using UCINET software [40].

**Figure 3:**
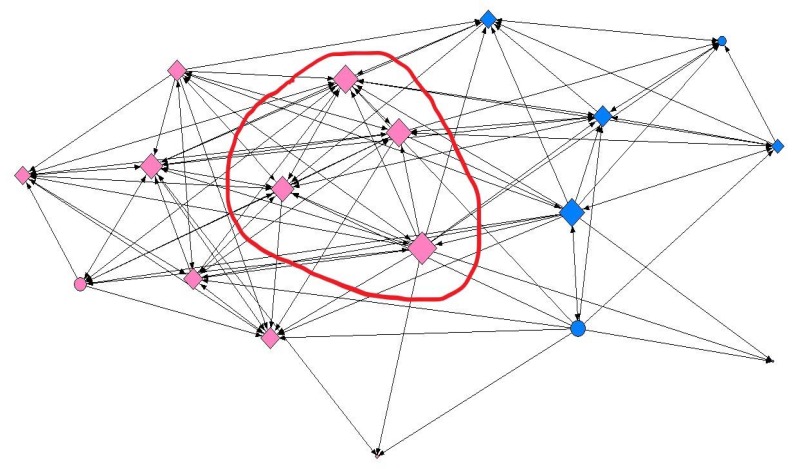
Graphical representation of one of the networks in the study at the intermediate contact intensity level, where node size indicates the degree of ties. Females are shown in pink, males in blue; circles represent individuals with a medium-low SES and diamonds a high SES. As can be seen, female adolescents with a high SES presented greatest centrality according to the degree of ties. Graphs were produced using UCINET software [40].

**Figure 4:**
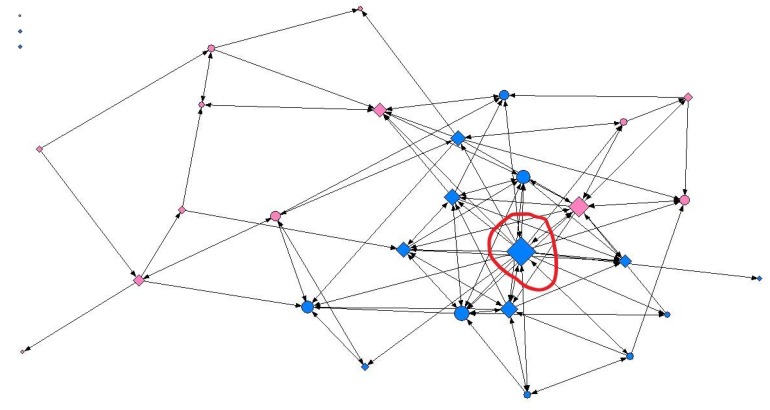
Graphical representation of one of the networks in the study at the maximum contact intensity level, where node size indicates the degree of ties. Females are shown in pink, males in blue; circles represent individuals with a medium-low SES and diamonds a high SES. As can be seen, male adolescents with a high SES presented greatest centrality according to the degree of ties. Graphs were produced using UCINET software [40].

Regarding reticular data, several density and centralization measures were calculated for each of the studied networks (Table 3). Additionally, the analysis of the social network indicated that overweight adolescents’ social ties did not reflect their SES at any of the contact intensity levels (Table 4, Table 5 and Table 6). In contrast, a statistical analysis of the overall sample irrespective of weight status revealed significant results at all three levels of contact intensity (Tables S1–S9). At the minimum contact level, adolescents with a high SES were almost twice as likely to present a greater capacity for intermediation (betweenness) (OR: 1.77; 95% CI: 1.001–3.148; *p* = 0.049), regardless of gender. By way of illustration, node size in Figure 2 represents the capacity for intermediation (betweenness) of adolescents in one of the social networks analyzed. As can be seen, larger nodes were predominantly associated with a high SES. At the intermediate contact level, we found differences by gender, more specifically in female adolescents. Thus, females with a high SES were more than twice as likely to be nominated as friends (indegree) (OR: 2.37; 95% CI: 1.022–5.518; *p* = 0.042) (Figure 3). At the maximum contact level, considered to represent friendship, we obtained similar results both for the overall sample and for male adolescents. We found that in general, the ease of establishing ties (outdegree) was two-fold higher in adolescents with a high SES (OR: 2.01; 95% CI: 1.126–3.588; *p* = 0.017). We obtained similar results for male adolescents (OR: 2.60; 95% CI: 1.139–5.962; *p* = 0.021) (Figure 4). 

## 4. Discussion

Our results showed that adolescents with a medium-low SES presented a higher probability of being overweight than those with a high SES. This agrees with several other studies that have reported an inverse relationship between SES and overweight, whereby the lower the SES, the higher the prevalence of overweight [6,8,11,41,42,43]. One explanation for this finding may be the influence SES exerts on the purchase and/or consumption of certain food products [12,13,44], for example, the high price of healthy products [43,45], the availability of grocery stores in certain neighborhoods offering a variety of products [43], or the possibility of eating homemade food [46]. However, other indicators such as parental educational level can also influence weight status by facilitating or restricting recommended information on this subject [44,46]. It has been shown that a low educational level in parents is related to the development of obesity [10,41,42]. Family structure (separated parents, single-parent family, large family, etc.) is another cultural factor that can influence the weight status of family members [47]. Similarly, the parenting styles according to the SES condition the existence of obesity [46,48]. Furthermore, the influence of the SES on the physical activity has also been proven, finding a greater level of physical activity when the SES is higher [15,49]. Aside from the role of the parents in this fact [50], the physical structure of certain neighborhoods, including their limitations, as the lack of recreational areas or playgrounds [43], the lack of appealing low-traffic zones with green areas [51], or the physical insecurity when practicing outdoor activities [43], can condition the level of physical exercise in the adolescent, according to his socioeconomic status. Nevertheless, other studies have obtained different results to those reported here. For example, Santos found a direct relationship between SES and overweight in Brazilian adolescents, whereby the higher the SES, the higher the prevalence of overweight [52], and Zhang, Zhao and Chu obtained the same relationship in an analysis of Chinese adolescents [53]. One possible explanation that has been suggested for this association is that young people in these countries have greater access to fast food restaurants and make greater use of computers and videogames, promoting obesity and physical inactivity, maybe related to the economic growth experienced by certain countries and the corresponding sociocultural changes. 

With respect to the relational data, little variations have been found regarding the centrality and density data in all the studied classrooms. This fact could be explained by the sociocentric perspective of the study, where each classroom is analyzed with a single system without having into account the existing contacts with other peers outside the class. Furthermore, we also found that overweight adolescents’ ties were not modified by their SES but instead were independent of their purchasing power. This fact leads us to think about the lack of homophilic characteristics in the overweight adolescent according to his SES, understanding homophily as the people’s preference for interacting with those with similar characteristics [54]. A priori, these results are consistent with the literature, since overweight and obese adolescents present greater difficulty in establishing ties and tend to be more isolated [55]. In light of our results, it seems that SES did not affect overweight adolescents’ willingness to establish contacts; it neither increased nor decreased their social capacity. We therefore deduce that it was their weight status which really determined their relational capacity. In contrast, other studies have found that purchasing power conditions social integration, with varying results according to the country where the study was conducted [56].

What our study has demonstrated, however, is that adolescents with a high SES present a significant trend towards socialization, irrespective of their weight status. At the minimum contact level, they occupied positions of intermediation, forming the necessary connections between the different groups established in the class. As the level of contact intensified (intermediate level), we found differences by gender. Female adolescents were more frequently nominated as friends, and thus had the opportunity to expand their social network by accepting these friendship ties. According to literature [57,58] it seems, then, that studied female adolescents are more prosocial regarding these friendship levels, being able to obtain more and greater resources from the network and protecting themselves from exclusion and isolation. At the friendship level, we found that all study participants with a high SES, but males in particular, were better positioned to form ties and therefore to establish support networks.

In this regard, authors such as Nieminen et al. have already noted the power of the social environment to condition norms and attitudes that modify behaviors aimed at improving health in general and self-esteem in particular [59]. Having a more extensive support network in adolescence implies having a resource that protects various aspects of health [60]. Difficulties in forming ties can be compounded by SES: individuals with few resources in the social structure are disadvantaged by their dependence on larger networks to access social resources [61], and in turn, the configuration of social networks is conditioned by socioeconomic status. Although networks vary over the course of life according to SES [62], the presence of these deficits at an early age restricts subsequent possibilities for improvement, thus contributing to greater inequality. 

## 5. Limitations

In this study, we used the FAS II questionnaire to measure participants’ SES. This decision could be seen as a limitation for the study, because the most widely used indicators in this respect are the social class, based on the more highly regarded occupation of the father or mother, and the parental educational level [4,46]. The reason for choosing this questionnaire as a SES indicator was, on the one hand, the design adapted to the adolescent population and, on the other, the fact that this questionnaire avoids questions concerning the SES of the parents, which participants may not know or not wish to answer. This way, we circumvented one of the difficulties posed by a study of this nature, namely whether data should be obtained from parents or students. Other limitations found in this work are the small sample size, as well as the lack of evaluation of other variables able to modify the capacity of establishing contacts with peers, and so the difficulty to extrapolate the results to different types of population. Also, the work has not taken into account relationships outside the classroom environment. Although studying the classroom as a social system itself is something inherent to the SNA from a sociocentric perspective, it also supposes an important limitation as contacts with other, external to the classrooms, groups are unknown to the study. 

## 6. Conclusions

Our study reveals the need to implement macro, meso and micro policies to combat the main problems that arise from having from a lower socioeconomic status (Table 7). In general, improving the life conditions since the early childhood stages, fighting for the equitable distribution of power and wealth, as well as recognizing the problem, measuring it and evaluating the results of the interventions are all necessary to tackle the problem effectively [63]. Equally important is the general public awareness about all the health determinants and especially this one [64]. We must, then, reconsider, and give constructive criticism, about the strategies set up at every action level, studying and analyzing what has been implemented and what has not, and keep working for an equitable community. This work highlights the social consequences of the SES at early ages, supposing this result a limitation regarding the establishment of the contact network and so a decrease in the acquisition of resources provided by the community. Having a low SES at an early age, indicates the need for actions that target children and adolescents, as well as adults, to reduce social inequalities [63,65]. It is, therefore, essential to improve the social networks of disadvantaged groups so as not to impede or hinder access to different resources precisely among those populations that need them most. Moreover, we have noticed a clear gap in the literature regarding this issue, what reveals a need for a greater amount of studies that can explore social networks and SES both in children and adolescents.

## Figures and Tables

**Table 1:** Composition scheme for the contact networks from the teaching classroom in each educational institution, and the corresponding participation rates.

Codified Institutions	Codified Networks	Number of Participants per Classroom	% Participation
COL1	Network1A	18	81.81%
	Network1B	19	86.36%
	Network1C	20	80.00%
COL2	Network2D	9	47.36%
COL3	Network3E	40	83.33%
	Network3F	29	70.73%
COL4	Network4G	51	64.55%
	Network4H	45	71.42%
COL5	Network5I	20	76.92%
	Network5J	15	51.72%
	Network5K	10	55.55%
	Total classrooms: 11	Total students: 276	

COL1–COL5: Representative code name for each participating educational institution in the study. Network1A–Network5K: Representative code name for each participating network in the study.

**Table 2:** Student distribution by participating network in the study.

Classroom	*N*	%
Network 1A	16	6.8
Network 1B	18	7.7
Network 1C	15	6.4
Network 2D	9	3.8
Network 3E	31	13.2
Network 3F	22	9.4
Network 4G	47	20
Network 4H	40	17
Network 5I	18	7.7
Network 5J	12	5.1
Network 5K	7	3.0
Total *N*	235	100

Network1A–Network5K: Representative code name for each participating network in the study.

**Table 3:** Density and centralization for each of the studied networks.

		Density	Centralization
Network1A	Minimum contact	0.708 ± 0.455	0.333
	Intermediate contact	0.275 ± 0.477	0.371
	Maximum contact	0.183 ± 0.387	0.095
Network1B	Minimum contact	0.729 ± 0.445	0.305
	Intermediate contact	0.392 ± 0.448	0.485
	Maximum contact	0.199 ± 0.400	0.371
Network1C	Minimum contact	0.657 ± 0.475	0.396
	Intermediate contact	0.214 ± 0.410	0.330
	Maximum contact	0.062 ± 0.241	0.176
Network2D	Minimum contact	0.903 ± 0.296	0.125
	Intermediate contact	0.542 ± 0.498	0.429
	Maximum contact	0.347 ± 0.476	0.518
Network3E	Minimum contact	0.701 ± 0.458	0.320
	Intermediate contact	0.259 ± 0.438	0.507
	Maximum contact	0.116 ± 0.320	0.517
Network3F	Minimum contact	0.634 ± 0.482	0.402
	Intermediate contact	0.249 ± 0.432	0.355
	Maximum contact	0.128 ± 0.334	0.174
Network4G	Minimum contact	0.547 ± 0.498	0.428
	Intermediate contact	0.211 ± 0.408	0.301
	Maximum contact	0.082 ± 0.275	0.345
Network4H	Minimum contact	0.563 ± 0.496	0.460
	Intermediate contact	0.221 ± 0.415	0.388
	Maximum contact	0.069 ± 0.253	0.144
Network5I	Minimum contact	0.627 ± 0.483	0.419
	Intermediate contact	0.239 ± 0.426	0.327
	Maximum contact	0.046 ± 0.209	0.147
Network5J	Minimum contact	0.606 ± 0.489	0.473
	Intermediate contact	0.220 ± 0.414	0.500
	Maximum contact	0.136 ± 0.343	0.491
Network5K	Minimum contact	0.833 ± 0.373	0.233
	Intermediate contact	0.524 ± 0.499	0.667
	Maximum contact	0.190 ± 0.393	0.667

Network1a–Network5k: Code names for the classrooms participating in the study.

**Table 4:** Estimation of probability of the relationship between SES in the overweight adolescent and network parameters, at the minimum contact intensity level.

Minimum Contact							
**SES**	**Low Outdegree**		**High Outdegree**		**OR**	**95% CI**	***p***
	***N***	**%**	***N***	**%**			
**Medium-low SES**	19	63.3	11	36.7	1		
**High SES**	24	58.5	17	41.5	1.22	0.46–3.22	0.683
	**Low Indegree**		**High Indegree**		**OR**	**95% CI**	***p***
	***N***	**%**	***N***	**%**			
**Medium-low SES**	13	43.3	17	56.7	1		
**High SES**	22	53.7	19	46.3	1.51	0.58–3.90	0.391
	**Low Degree**		**High Degree**		**OR**	**95% CI**	***p***
	***N***	**%**	***N***	**%**			
**Medium-low SES**	16	53.3	14	46.7	1		
**High SES**	21	51.2	20	48.8	1.08	0.42–2.79	0.860
	**Low Incloseness**		**High Incloseness**		**OR**	**95% CI**	***p***
	***N***	**%**	***N***	**%**			
**Medium-low SES**	13	43.3	17	56.7	1		
**High SES**	22	53.7	19	46.3	1.51	0.58–3.90	0.391
	**Low Outcloseness**		**High Outcloseness**		**OR**	**95% CI**	***p***
	***N***	**%**	***N***	**%**			
**Medium-low SES**	19	63.3	11	36.7	1		
**High SES**	24	58.5	17	41.5	1.22	0.46–3.22	0.683
	**Low Betweenness**		**High Betweenness**		**OR**	**95% CI**	***p***
	***N***	**%**	***N***	**%**			
**Medium-low SES**	19	63.3	11	36.7	1		
**High SES**	20	48.8	21	51.2	1.81	0.69–4.74	0.223
	**Low Eigenvector**		**High Eigenvector**		**OR**	**95% CI**	***p***
	***N***	**%**	***N***	**%**			
**Medium-low SES**	14	46.7	16	53.3	1		
**High SES**	22	53.7	19	46.3	1.32	0.51–3.40	0.561

Outdegree: nominations emitted by the individual; indegree: nominations received by the individual; degree: relational capacity; in/outcloseness: individual’s proximity to the rest of the network; betweenness: capacity for intermediation; eigenvector: prestige/influence.

**Table 5:** Estimation of probability of the relationship between SES in the overweight adolescent and network parameters, at the intermediate contact intensity level.

Intermediate Contact							
**SES**	**Low Outdegree**		**High Outdegree**		**OR**	**95% CI**	***p***
	***N***	**%**	***N***	**%**			
**Medium-low SES**	19	63.3	11	36.7	1		
**High SES**	24	58.5	17	41.5	1.22	0.46–3.22	0.683
	**Low Indegree**		**High Indegree**		**OR**	**95% CI**	***p***
	***N***	**%**	***N***	**%**			
**Medium-low SES**	18	60	12	40	1		
**High SES**	22	53.7	19	46.3	1.29	0.49–3.36	0.595
	**Low Degree**		**High Degree**		**OR**	**95% CI**	***p***
	***N***	**%**	***N***	**%**			
**Medium-low SES**	15	50	15	50	1		
**High SES**	18	43.9	23	56.1	1.27	0.49–3.28	0.611
	**Low Incloseness**		**High Incloseness**		**OR**	**95% CI**	***p***
	***N***	**%**	***N***	**%**			
**Medium-low SES**	16	53.3	14	46.7	1		
**High SES**	14	34.1	27	65.9	2.20	0.84–5.78	0.106
	**Low Outcloseness**		**High Outcloseness**		**OR**	**95% CI**	***p***
	***N***	**%**	***N***	**%**			
**Medium-low SES**	17	56.7	13	43.3	1		
**High SES**	22	53.7	19	46.3	1.12	0.43–2.91	0.801
	**Low Betweenness**		**High Betweenness**		**OR**	**95% CI**	***p***
	***N***	**%**	***N***	**%**			
**Medium-low SES**	20	66.7	10	33.3	1		
**High SES**	20	48.8	21	51.2	2.10	0.79–5.56	0.133
	**Low Eigenvector**		**High Eigenvector**		**OR**	**95% CI**	***p***
	***N***	**%**	***N***	**%**			
**Medium-low SES**	18	60	12	40	1		
**High SES**	20	48.8	21	51.2	1.57	0.60–4.08	0.349

Outdegree: nominations emitted by the individual; indegree: nominations received by the individual; degree: relational capacity; in/outcloseness: individual’s proximity to the rest of the network; betweenness: capacity for intermediation; eigenvector: prestige/influence.

**Table 6:** Estimation of probability in an analysis of the relationship between SES in the overweight adolescent and network parameters, at the maximum contact intensity level

Maximum Contact							
**SES**	**Low Outdegree**		**High Outdegree**		**OR**	**95% CI**	***p***
	***N***	**%**	***N***	**%**			
**Medium-low SES**	18	60	12	40	1		
**High SES**	20	48.8	21	51.2	1.57	0.60–4.08	0.349
	**Low Indegree**		**High Indegree**		**OR**	**95% CI**	***p***
	***N***	**%**	***N***	**%**			
**Medium-low SES**	13	43.3	17	56.7	1		
**High SES**	22	53.7	19	46.3	1.51	0.58–3.90	0.390
	**Low Degree**		**High Degree**		**OR**	**95% CI**	***p***
	***N***	**%**	***N***	**%**			
**Medium-low SES**	15	50	15	50	1		
**High SES**	20	48.8	21	51.2	1.05	0.40–2.69	0.919
	**Low Incloseness**		**High Incloseness**		**OR**	**95% CI**	***p***
	***N***	**%**	***N***	**%**			
**Medium-low SES**	13	43.3	17	56.7	1		
**High SES**	21	51.2	20	48.8	1.37	0.53–3.53	0.511
	**Low Outcloseness**		**High Outcloseness**		**OR**	**95% CI**	***p***
	***N***	**%**	***N***	**%**			
**Medium-low SES**	16	53.3	14	46.7	1		
**High SES**	19	46.3	22	53.7	1.32	0.51–3.40	0.561
	**Low Betweenness**		**High Betweenness**		**OR**	**95% CI**	***p***
	***N***	**%**	***N***	**%**			
**Medium-low SES**	17	56.7	13	43.3			
**High SES**	20	48.8	21	51.2	1.37	0.53–3.53	0.511
	**Low Eigenvector**		**High Eigenvector**		**OR**	**95% CI**	***p***
	***N***	**%**	***N***	**%**			
**Medium-low SES**	15	50	15	50	1		
**High SES**	21	51.2	20	48.8	1.05	0.40–2.69	0.919

Outdegree: nominations emitted by the individual; indegree: nominations received by the individual; degree: relational capacity; in/outcloseness: individual’s proximity to the rest of the network; betweenness: capacity for intermediation; eigenvector: prestige/influence.

**Table 7:** Suggestion for improvement at the macro, meso and micro action levels.

Macro Levels (Governments and International Agencies)	Comprehensive approaches that ensure the principles of child development (physical, cognitive, social and emotional) Ensure the development and economic growth of the country Promotion and implementation of social policies Meet the needs of rural communities Employment policies that guarantee a balance between work and family life Guarantee social protection in vulnerable periods of life (illness, disability or unemployment) Universal health based on primary care, accessible to all citizens regardless of their SES Promotion of affordable housing policies National and international regulation policies for the marketing of little or no healthy products Government tax reform policies that encourage health promotion activities Debt relief for certain countries
Meso Levels (Community)	Planning and design of urban environments aimed at improving physical and psychological well-being Improvement of marginal neighborhoods Improvement of employment opportunities Promotion of participation in health activities Design of specific educational programs dedicated to the problem Involvement of all members of the community
Micro Levels (Individual)	Addressing the problem centered on the person Individualized assistance to modify lifestyles Psychosocial care Improvement of the level of training from the infant stages, also involving parents and caregivers Empowerment in one’s health management

Own compilation based on literature [63,66,67]

## Supplementary Materials

The following are available online at http://www.mdpi.com/1660-4601/15/9/2014/s1, Table S1: Estimation of probability of the relationship between SES in the overall sample and network parameters, at the minimum contact intensity level. Table S2: Estimation of probability of the relationship between SES in the overall sample and network parameters, at the intermediate contact intensity level. Table S3: Estimation of probability of an analysis of the relationship between SES in the overall sample and network parameters, at the maximum contact intensity level. Table S4: Estimation of probability of an analysis of the relationship between SES in the female gender of the overall sample and network parameters, at the minimum contact intensity level. Table S5: Estimation of probability of an analysis of the relationship between SES in the female gender of the overall sample and network parameters, at the intermediate contact intensity level. Table S6: Estimation of probability of an analysis of the relationship between SES in the female gender of the overall sample and network parameters, at the maximum contact intensity level. Table S7: Estimation of probability of an analysis of the relationship between SES in the male gender of the overall sample and network parameters, at the minimum contact intensity level. Table S8: Estimation of probability of an analysis of the relationship between SES in the male gender of the overall sample and network parameters, at the intermediate contact intensity level. Table S9: Estimation of probability of an analysis of the relationship between SES in the male gender of the overall sample and network parameters, at the maximum contact intensity level.
