# Supplementary material for: Socioeconomic Status in Adolescents: A Study of Its Relationship with Overweight and Obesity and Influence on Social Network Configuration
Source: Int J Environ Res Public Health. 2018 Sep 15;15(9):2014. doi: 10.3390/ijerph15092014 (PMC6163670; doi:10.3390/ijerph15092014)
Supplement: Supplementary file 1 [file ijerph-15-02014-s001.pdf]

Table S1. Estimation of probability of the relationship between SES in the overall sample and network parameters, at the minimum contact intensity level.

Minimum Contact							
	**Low Outdegree**		**High Outdegree**		**OR**	**95% CI**	***p***
	N	%	*N*	%			
**Medium-low SES**	40	58.8	28	41.2	1		
**High SES**	78	46.7	89	53.3	1.63	0.92–2.88	0.092
	**Low Indegree**		**High Indegree**		**OR**	**95% CI**	***p***
	N	%	*N*	%			
**Medium-low SES**	37	54.4	31	45.6	1		
**High SES**	81	48.5	86	51.5	1.26	0.72–2.23	0.411
	**Low Degree**		**High Degree**		**OR**	**95% CI**	***p***
	N	%	*N*	%			
**Medium-low SES**	36	52.9	32	47.1	1		
**High SES**	82	49.1	85	50.9	1.16	0.66–2.05	0.593
	**Low Incloseness**		**High Incloseness**		**OR**	**95% CI**	***p***
	N	%	*N*	%			
**Medium-low SES**	37	54.4	31	45.6	1		
**High SES**	83	49.7	84	50.3	1.20	0.68–2.12	0.512
	**Low Outcloseness**		**High Outcloseness**		**OR**	**95% CI**	***p***
	N	%	*N*	%			
**Medium-low SES**	40	58.8	28	41.2	1		
**High SES**	78	46.7	89	53.3	1.63	0.92–2.88	0.092
	**Low Betweenness**		**High Betweenness**		**OR**	**95% CI**	***p***
	N	%	*N*	%			
**Medium-low SES**	41	60.3	27	39.7	1		
**High SES**	77	46.1	90	53.9	1.77	1.00–3.14	**0.049**
	**Low Eigenvector**		**High Eigenvector**		**OR**	**95% CI**	***p***
	N	%	*N*	%			
**Medium-low SES**	36	52.9	32	47.1	1		
**High SES**	82	49.1	85	50.9	1.16	0.66–2.05	0.593

Outdegree: nominations emitted by the individual; indegree: nominations received by the individual; degree: relational capacity; in/outcloseness: individual's proximity to the rest of the network; betweenness: capacity for intermediation; eigenvector: prestige/influence.

Table S2. Estimation of probability of the relationship between SES in the overall sample and network parameters, at the intermediate contact intensity level.

Intermediate Contact							
	**Low Outdegree**		**High Outdegree**		**OR**	**95% CI**	***p***
	N	%	*N*	%			
**Medium-low SES**	40	58.8	28	41.2	1		
**High SES**	78	46.7	89	53.3	1.63	0.92–2.88	0.092
	**Low Indegree**		**High Indegree**		**OR**	**95% CI**	***p***
	N	%	*N*	%			
**Medium-low SES**	40	58.8	28	41.2	1		
**High SES**	80	47.9	87	52.1	1.55	0.87–2.74	0.129
	**Low Degree**		**High Degree**		**OR**	**95% CI**	***p***
	N	%	*N*	%			
**Medium-low SES**	36	52.9	32	47.1	1		
**High SES**	84	50.3	83	49.7	1.11	0.63–1.95	0.713
	**Low Incloseness**		**High Incloseness**		**OR**	**95% CI**	***p***
	N	%	*N*	%			
**Medium-low SES**	38	55.9	30	44.1	1		
**High SES**	81	48.5	86	51.5	1.34	0.76–2.37	0.305

	Low Outcloseness		High Outcloseness		OR	95% CI	*p*
	N	%	*N*	%			
**Medium-low SES**	37	54.4	31	45.6	1		
**High SES**	81	48.5	86	51.5	1.26	0.72–2.23	0.411
	**Low Betweenness**		**High Betweenness**		**OR**	**95% CI**	***p***
	N	%	*N*	%			
**Medium-low SES**	39	57.4	29	42.6	1		
**High SES**	79	47.3	88	52.7	1.49	0.84–2.64	0.162
	**Low Eigenvector**		**High Eigenvector**		**OR**	**95% CI**	***p***
	N	%	*N*	%			
**Medium-low SES**	39	57.4	29	42.6			
**High SES**	80	47.9	87	52.1	1.46	0.82–2.58	0.189

Outdegree: nominations emitted by the individual; indegree: nominations received by the individual; degree: relational capacity; in/outcloseness: individual's proximity to the rest of the network; betweenness: capacity for intermediation; eigenvector: prestige/influence.

Table S3. Estimation of probability of an analysis of the relationship between SES in the overall sample and network parameters, at the maximum contact intensity level.

	Maximum Contact						
	**Low Outdegree**		**High Outdegree**		**OR**	**95% CI**	***p***
	N	%	*N*	%			
**Medium-low SES**	43	63.2	25	36.8	1		
**High SES**	77	46.1	90	53.9	2.01	1.12–3.58	**0.017**
	**Low Indegree**		**High Indegree**		**OR**	**95% CI**	***p***
	N	%	*N*	%			
**Medium-low SES**	32	47.1	36	52.9	1		
**High SES**	90	53.9	77	46.1	0.76	0.43–1.33	0.342
	**Low Degree**		**High Degree**		**OR**	**95% CI**	***p***
	N	%	*N*	%			
**Medium-low SES**	36	52.9	32	47.1	1		
**High SES**	85	50.9	82	49.1	1.08	0.61–1.90	0.776
	**Low Incloseness**		**High Incloseness**		**OR**	**95% CI**	***p***
	N	%	*N*	%			
**Medium-low SES**	29	42.6	39	57.4	1		
**High SES**	89	53.3	78	46.7	0.65	0.36–1.15	0.139
	**Low Outcloseness**		**High Outcloseness**		**OR**	**95% CI**	***p***
	N	%	*N*	%			
**Medium-low SES**	39	57.4	29	42.6	1		
**High SES**	81	48.5	86	51.5	1.42	0.80–2.52	0.218
	**Low Betweenness**		**High Betweenness**		**OR**	**95% CI**	***p***
	N	%	*N*	%			
**Medium-low SES**	40	58.8	28	41.2	1		
**High SES**	78	46.7	89	53.3	1.63	0.92–2.88	0.092
	**Low Eigenvector**		**High Eigenvector**		**OR**	**95% CI**	***p***
	N	%	*N*	%			
**Medium-low SES**	38	55.9	30	44.1	1		
**High SES**	80	47.9	87	52.1	1.37	0.78–2.42	0.267

Outdegree: nominations emitted by the individual; indegree: nominations received by the individual; degree: relational capacity; in/outcloseness: individual's proximity to the rest of the network; betweenness: capacity for intermediation; eigenvector: prestige/influence.

Table S4. Estimation of probability of an analysis of the relationship between SES in the female gender of the overall sample and network parameters, at the minimum contact intensity level.

Minimum Contact							
	Low Outdegree		High Outdegree		OR	95% CI	*p*
	N	%	*N*	%			
**Medium-low SES**	17	54.8	14	45.2	1		
**High SES**	38	44.7	47	55.3	1.50	0.65–3.43	0.333
	**Low Indegree**		**High Indegree**		**OR**	**95% CI**	***p***
	N	%	*N*	%			
**Medium-low SES**	18	58.1	13	41.9	1		
**High SES**	40	47.1	45	52.9	1.55	0.67–3.57	0.294
	**Low Degree**		**High Degree**		**OR**	**95% CI**	***p***
	N	%	*N*	%			
**Medium-low SES**	18	58.1	13	41.9	1		
**High SES**	41	48.2	44	51.8	1.48	0.64–3.41	0.349
	**Low Incloseness**		**High Incloseness**		**OR**	**95% CI**	***p***
	N	%	*N*	%			
**Medium-low SES**	18	58.1	13	41.9	1		
**High SES**	39	45.9	46	54.1	1.63	0.71–3.75	0.245
	**Low Outcloseness**		**High Outcloseness**		**OR**	**95% CI**	***p***
	N	%	*N*	%			
**Medium-low SES**	17	54.8	14	45.2	1		
**High SES**	38	44.7	47	55.3	1.50	0.65–3.43	0.333
	**Low Betweenness**		**High Betweenness**		**OR**	**95% CI**	***p***
	N	%	*N*	%			
**Medium-low SES**	19	61.3	12	38.7	1		
**High SES**	40	47.1	45	52.9	1.78	0.77–4.12	0.175
	**Low Eigenvector**		**High Eigenvector**		**OR**	**95% CI**	***p***
	N	%	*N*	%			
**Medium-low SES**	18	58.1	13	41.9	1		
**High SES**	42	49.4	43	50.6	1.41	0.61–3.25	0.409

Outdegree: nominations emitted by the individual; indegree: nominations received by the individual; degree: relational capacity; in/outcloseness: individual's proximity to the rest of the network; betweenness: capacity for intermediation; eigenvector: prestige/influence.

Table S5. Estimation of probability of an analysis of the relationship between SES in the female gender of the overall sample and network parameters, at the intermediate contact intensity level.

Intermediate Contact							
	Low Outdegree		High Outdegree		OR	95% CI	*p*
	N	%	*N*	%			
**Medium-low SES**	19	61.3	12	38.7	1		
**High SES**	39	45.9	46	54.1	1.86	0.80–4.32	0.142
	**Low Indegree**		**High Indegree**		**OR**	**95% CI**	***p***
	N	%	*N*	%			
**Medium-low SES**	19	61.3	12	38.7	1		
**High SES**	34	40	51	60	2.37	1.02–5.51	**0.042**
	**Low Degree**		**High Degree**		**OR**	**95% CI**	***p***
	N	%	*N*	%			
**Medium-low SES**	18	58.1	13	41.9	1		
**High SES**	46	54.1	39	45.9	1.17	0.51–2.69	0.705
	**Low Incloseness**		**High Incloseness**		**OR**	**95% CI**	***p***
	N	%	*N*	%			
**Medium-low SES**	17	54.8	14	45.2	1		
**High SES**	37	43.5	48	56.5	1.57	0.68–3.60	0.280

	Low Outcloseness		High Outcloseness		OR	95% CI	*p*
	N	%	*N*	%			
**Medium-low SES**	18	58.1	13	41.9	1		
**High SES**	41	48.2	44	51.8	1.48	0.64–3.41	0.349
	**Low Betweenness**		**High Betweenness**		**OR**	**95% CI**	***p***
	N	%	*N*	%			
**Medium-low SES**	18	58.1	13	41.9	1		
**High SES**	36	42.4	49	57.6	1.88	0.81–4.33	0.133
	**Low Eigenvector**		**High Eigenvector**		**OR**	**95% CI**	***p***
	N	%	*N*	%			
**Medium-low SES**	19	61.3	12	38.7	1		
**High SES**	44	51.8	41	48.2	1.47	0.63–3.41	0.362

Outdegree: nominations emitted by the individual; indegree: nominations received by the individual; degree: relational capacity; in/outcloseness: individual's proximity to the rest of the network; betweenness: capacity for intermediation; eigenvector: prestige/influence.

Table S6. Estimation of probability of an analysis of the relationship between SES in the female gender of the overall sample and network parameters, at the maximum contact intensity level.

	Maximum Contact						
	**Low Outdegree**		**High Outdegree**		**OR**	**95% CI**	***p***
	N	%	*N*	%			
**Medium-low SES**	17	54.8	14	45.2	1		
**High SES**	38	44.7	47	55.3	1.50	0.65–3.43	0.333
	**Low Indegree**		**High Indegree**		**OR**	**95% CI**	***p***
	N	%	*N*	%			
**Medium-low SES**	13	41.9	18	58.1	1		
**High SES**	41	48.2	44	51.8	0.77	0.33–1.77	0.547
	**Low Degree**		**High Degree**		**OR**	**95% CI**	***p***
	N	%	*N*	%			
**Medium-low SES**	17	54.8	14	45.2	1		
**High SES**	45	52.9	40	47.1	1.07	0.47–2.46	0.856
	**Low Incloseness**		**High Incloseness**		**OR**	**95% CI**	***p***
	N	%	*N*	%			
**Medium-low SES**	12	38.7	19	61.3	1		
**High SES**	48	56.5	37	43.5	0.48	0.21–1.12	0.090
	**Low Outcloseness**		**High Outcloseness**		**OR**	**95% CI**	***p***
	N	%	*N*	%			
**Medium-low SES**	19	61.3	12	38.7	1		
**High SES**	41	48.2	44	51.8	1.69	0.73–3.93	0.213
	**Low Betweenness**		**High Betweenness**		**OR**	**95% CI**	***p***
	N	%	*N*	%			
**Medium-low SES**	17	54.8	14	45.2	1		
**High SES**	39	45.9	46	54.1	1.43	0.62–3.27	0.393
	**Low Eigenvector**		**High Eigenvector**		**OR**	**95% CI**	***p***
	N	%	*N*	%			
**Medium-low SES**	17	54.8	14	45.2	1		
**High SES**	41	48.2	44	51.8	1.30	0.57–2.97	0.529

Outdegree: nominations emitted by the individual; indegree: nominations received by the individual; degree: relational capacity; in/outcloseness: individual's proximity to the rest of the network; betweenness: capacity for intermediation; eigenvector: prestige/influence.

Table S7. Estimation of probability of an analysis of the relationship between SES in the male gender of the overall sample and network parameters, at the minimum contact intensity level.

	Minimum Contact						
	Low Outdegree		High Outdegree		OR	95% CI	*p*
	N	%	*N*	%			
**Medium-low SES**	23	62.2	14	37.8	1		
**High SES**	40	48.8	42	51.2	1.72	0.78–3.81	0.176
	**Low Indegree**		**High Indegree**		**OR**	**95% CI**	*p*
	N	%	*N*	%			
**Medium-low SES**	19	51.4	18	48.6	1		
**High SES**	41	50	41	50	1.05	0.48–2.29	0.891
	**Low Degree**		**High Degree**		**OR**	**95% CI**	*p*
	N	%	*N*	%			
**Medium-low SES**	18	48.6	19	51.4	1		
**High SES**	41	50	41	50	0.94	0.43–2.05	0.891
	**Low Incloseness**		**High Incloseness**		**OR**	**95% CI**	*p*
	N	%	*N*	%			
**Medium-low SES**	19	51.4	18	48.6	1		
**High SES**	44	53.7	38	46.3	0.91	0.41–1.98	0.815
	**Low Outcloseness**		**High Outcloseness**		**OR**	**95% CI**	*p*
	N	%	*N*	%			
**Medium-low SES**	23	62.2	14	37.8	1		
**High SES**	40	48.8	42	51.2	1.72	0.78–3.81	0.176
	**Low Betweenness**		**High Betweenness**		**OR**	**95% CI**	*p*
	N	%	*N*	%			
**Medium-low SES**	22	59.5	15	40.5	1		
**High SES**	37	45.1	45	54.9	1.78	0.81–3.92	0.148
	**Low Eigenvector**		**High Eigenvector**		**OR**	**95% CI**	*p*
	N	%	*N*	%			
**Medium-low SES**	18	48.6	19	51.4	1		
**High SES**	40	48.8	42	51.2	0.99	0.45–2.16	0.989

Outdegree: nominations emitted by the individual; indegree: nominations received by the individual; degree: relational capacity; in/outcloseness: individual's proximity to the rest of the network; betweenness: capacity for intermediation; eigenvector: prestige/influence.

Table S8. Estimation of probability of an analysis of the relationship between SES in the male gender of the overall sample and network parameters, at the intermediate contact intensity level.

	Intermediate Contact						
	Low Outdegree		High Outdegree		OR	95% CI	*p*
	N	%	*N*	%			
**Medium-low SES**	21	56.8	16	43.2	1		
**High SES**	39	47.6	43	52.4	1.44	0.66–3.16	0.353
	**Low Indegree**		**High Indegree**		**OR**	**95% CI**	*p*
	N	%	*N*	%			
**Medium-low SES**	21	56.8	16	43.2	1		
**High SES**	46	56.1	36	43.9	1.02	0.46–2.24	0.946
	**Low Degree**		**High Degree**		**OR**	**95% CI**	*p*
	N	%	*N*	%			
**Medium-low SES**	18	48.6	19	51.4	1		
**High SES**	38	46.3	44	53.7	1.09	0.50–2.38	0.815
	**Low Incloseness**		**High Incloseness**		**OR**	**95% CI**	*p*
	N	%	*N*	%			
**Medium-low SES**	21	56.8	16	43.2	1		
**High SES**	44	53.7	38	46.3	1.13	0.51–2.47	0.753

	Low Outcloseness		High Outcloseness		OR	95% CI	*p*
	N	%	*N*	%			
**Medium-low SES**	19	51.4	18	48.6	1		
**High SES**	40	48.8	42	51.2	1.10	0.51–2.41	0.795
	**Low Betweenness**		**High Betweenness**		**OR**	**95% CI**	***p***
	N	%	*N*	%			
**Medium-low SES**	21	56.8	16	43.2	1		
**High SES**	43	52.4	39	47.6	1.19	0.54–2.60	0.662
	**Low Eigenvector**		**High Eigenvector**		**OR**	**95% CI**	***p***
	N	%	*N*	%			
**Medium-low SES**	20	54.1	17	45.9	1		
**High SES**	36	43.9	46	56.1	1.50	0.68–3.27	0.304

Outdegree: nominations emitted by the individual; indegree: nominations received by the individual; degree: relational capacity; in/outcloseness: individual's proximity to the rest of the network; betweenness: capacity for intermediation; eigenvector: prestige/influence.

Table S9. Estimation of probability of an analysis of the relationship between SES in the male gender of the overall sample and network parameters, at the maximum contact intensity level.

	Maximum Contact						
	**Low Outdegree**		**High Outdegree**		**OR**	**95% CI**	***p***
	N	%	*N*	%			
**Medium-low SES**	26	70.3	11	29.7	1		
**High SES**	39	47.6	43	52.4	2.60	1.13–5.96	**0.021**
	**Low Indegree**		**High Indegree**		**OR**	**95% CI**	***p***
	N	%	*N*	%			
**Medium-low SES**	19	51.4	18	48.6	1		
**High SES**	49	59.8	33	40.2	0.71	0.32–1.55	0.391
	**Low Degree**		**High Degree**		**OR**	**95% CI**	***p***
	N	%	*N*	%			
**Medium-low SES**	19	51.4	18	48.6	1		
**High SES**	40	48.8	42	51.2	1.10	0.51–2.41	0.795
	**Low Incloseness**		**High Incloseness**		**OR**	**95% CI**	***p***
	N	%	*N*	%			
**Medium-low SES**	17	45.9	20	54.1	1		
**High SES**	41	50	41	50	0.85	0.39–1.85	0.682
	**Low Outcloseness**		**High Outcloseness**		**OR**	**95% CI**	***p***
	N	%	*N*	%			
**Medium-low SES**	20	54.1	17	45.9	1		
**High SES**	40	48.8	42	51.2	1.23	0.56–2.69	0.594
	**Low Betweenness**		**High Betweenness**		**OR**	**95% CI**	***p***
	N	%	*N*	%			
**Medium-low SES**	23	62.2	14	37.8	1		
**High SES**	39	47.6	43	52.4	1.81	0.81–4.00	0.140
	**Low Eigenvector**		**High Eigenvector**		**OR**	**95% CI**	***p***
	N	%	*N*	%			
**Medium-low SES**	21	56.8	16	43.2	1		
**High SES**	39	47.6	43	52.4	1.44	0.66–3.16	0.353

Outdegree: nominations emitted by the individual; indegree: nominations received by the individual; degree: relational capacity; in/outcloseness: individual's proximity to the rest of the network; betweenness: capacity for intermediation; eigenvector: prestige/influence.
